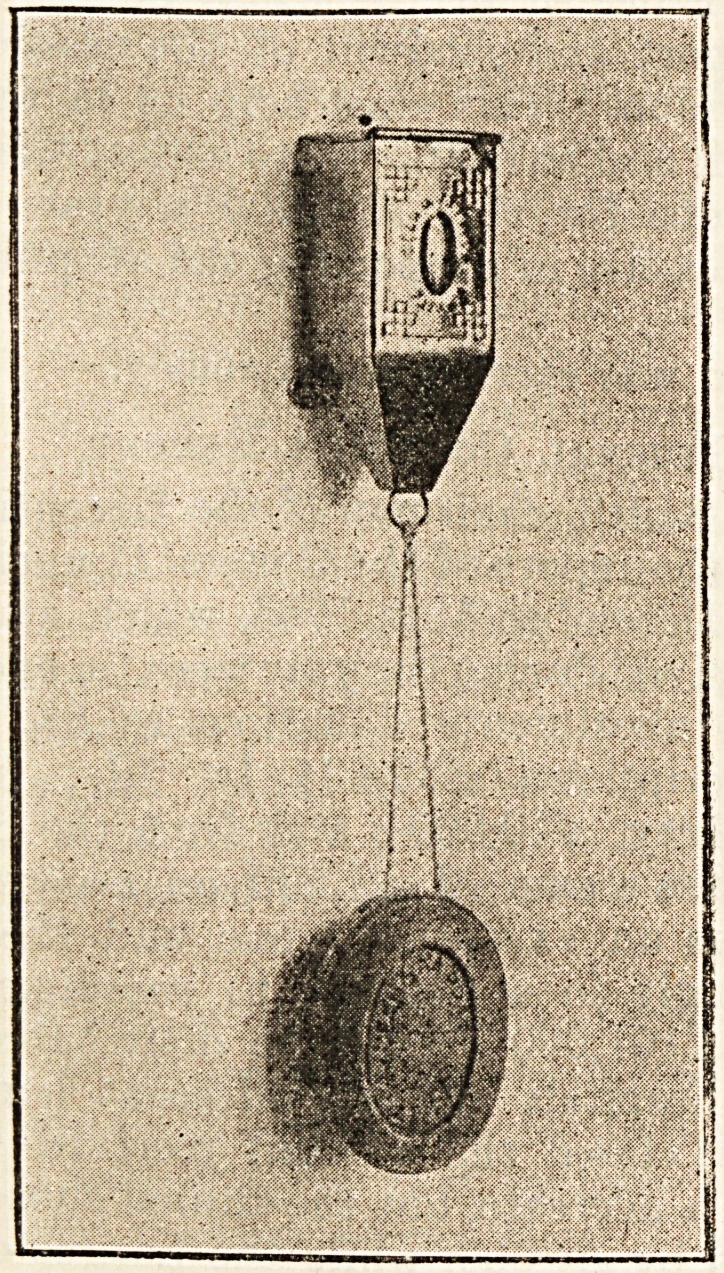# New Appliances and Things Medical

**Published:** 1904-03-12

**Authors:** 


### NEW APPLIANCES AND THINGS MEDICAL.

[We shall be glad to receive at our Office, 28 & 29 Southampton Street, Strand, London, W.C., from the manufacturers, specimens of all new preparations and appliances which may be brought out from time to time.]

### PATENT SOAP TIDY.

MESSRS. DEAN AND FARRAR, of Halifax, have sent us a contrivance for the use of soap which is both economical and cleanly. The illustration shows that the soap is retained free from contact with water in the soap dish by means of a string attachment to a spring pulley. The return of the soap into posit[illegible]n after use in the basin is easy and mechanical and causes [illegible] trouble. In hospitals and surgeries it is certainly to be rec[illegible]mmended as a suitable manner of identifying the soap to be kep[illegible] for the use of medical men.

### EM'S MEDICATED NASAL WOOL.

THIS preparation, which is sold under the name of "Naswol," consists of absorbent wool charged with eucalyptus, terebene, and menthol. It provides a convenient and easy method of applying these drugs to the upper respiratory passages. The preparation can be employed with advantage in conditions where the nasal mucosa is congested, as in hay fever and the so-called "cold in the head." A small portion of the wool is inserted in the nostrils, and air inhaled through it. In this way the volatile drugs contained are carried to the mucous membrane of the nasopharynx, where they exert a two-fold action by relieving the local congestion, by causing the blood-vessels to contract, and applying antiseptics to the infected surface. We have put this medicated wool to practical tests on patients suffering from acute rhinitis, and, as might be expected, they have experienced well-marked temporary, if not permanent, relief. Certainly it provides a handier means of applying the drugs contained than those commonly in use, such as inhaling the steam from hot water to which the volatile drugs have been added.

### PROTENE PREPARATIONS.

(THE PROTENE COMPANY, LIMITED, 36 WELBECK STREET, CAVENDISH SQUARE, LONDON, W.)

PROTENE and many preparations containing it have been now before the public for some years. Their nutritive qualities, which are of a high order, and their concentrated form, together with their ready assimilation, have supplied a distinct want, so that we heartily welcome additional preparations which will extend their usefulness to a still wider section of the community. "Protene bran bread" is one of these new productions. It constitutes a great advance on the poorly nutritive breads hitherto provided for diabetics. Containing as it does a high percentage of proteid and a fair proportion of fat, with practically an entire absence of carbohydrate material, it is an ideal bread for those afflicted with diabetes. The addition of the bran is to counteract any tendency to constipation that may exist. The "Alma" biscuit is specially intended for use in cases where the carbohydrate in the diet must be kept low but not avoided altogether, such as in cases of obesity, gouty conditions, etc. The "Welbeck" biscuit containing 25 per cent. of proteid is most palatable, and intended for general use. "Protene Cocoa" forms a pleasant beverage, and, being highly nutritious as well, is specially adapted for the use of invalids and convalescents.

## Figures and Tables

**Figure f1:**